# A complete year of urology residency training under COVID-19: impact on education and health

**DOI:** 10.1590/S1677-5538.IBJU.2024.0240

**Published:** 2024-07-20

**Authors:** João Victor T. Henriques, José A. Prezotti, Karin M. Anzolch, Gustavo Ruschi, Gilberto Almeida, Leonardo Seligra, Luciano A. Favorito, Alfredo F. Canalini, Roni de C. Fernandes, Fransber R. A. Rodrigues, Caroline Santos Silva, Anna Sophia Candiotto Pereira, José de Bessa, Cristiano M. Gomes

**Affiliations:** 1 Faculdade de Medicina da Universidade de São Paulo Divisão de Urologia São Paulo SP Brasil Divisão de Urologia, Faculdade de Medicina da Universidade de São Paulo - FMUSP, São Paulo, SP, Brasil; 2 Hospital Moinhos de Vento Porto Alegre RS Brasil Serviço de Urologia, Hospital Moinhos de Vento, Porto Alegre, RS, Brasil; 3 Universidade Federal do Espírito Santo Vitória ES Brasil Universidade Federal do Espírito Santo – UFES, Vitória, ES, Brasil; 4 Universidade do Vale do Itajaí Itajaí SC Brasil Universidade do Vale do Itajaí – UNIVALE, Itajaí, SC, Brasil; 5 Universidade Federal do ABC Santo André SP Brasil Disciplina de Urologia, Universidade Federal do ABC - UFABC, Santo André, SP, Brasil; 6 Universidade do Estado do Rio de Janeiro Unidade de Pesquisa Urogenital Rio de Janeiro RJ Brasil Unidade de Pesquisa Urogenital, Universidade do Estado do Rio de Janeiro - UERJ, Rio de Janeiro, Rio de Janeiro, RJ, Brasil; 7 Universidade do Estado do Rio de Janeiro Rio de Janeiro RJ Brasil Disciplina de Urologia, Universidade do Estado do Rio de Janeiro - UERJ, Rio de Janeiro, Rio de Janeiro, RJ, Brasil; 8 Santa Casa de São Paulo Faculdade de Ciências Médicas São Paulo SP Brasil Faculdade de Ciências Médicas, Santa Casa de São Paulo, São Paulo, SP, Brasil; 9 Universidade de Brasília Divisão de Urologia Brasília DF Brasil Divisão de Urologia, Universidade de Brasília - UNB, Brasília, DF, Brasil; 10 Universidade Estadual de Feira de Santana Departamento de Cirurgia Feira de Santana BA Brasil Departamento de Cirurgia, Universidade Estadual de Feira de Santana, Feira de Santana, BA, Brasil; 11 Instituto de Pesquisa, Gestão e Tecnologia Belo Horizonte MG Brasil Núcleo Técnico. Instituto de Pesquisa, Gestão e Tecnologia – INTEC. Belo Horizonte, MG, Brasil

**Keywords:** COVID-19, Education, Medical, Surveys and Questionnaires, Brazil

## Abstract

**Objectives:**

To evaluate the impact of COVID-19 pandemics on clinical and surgical practice, educational activities, health and lifestyle behavior of Brazilian urology residents after 1 year of socio-economic restrictions.

**Materials and Methods:**

An electronic survey was e-mailed to all postgraduate (PG) students registered by the Brazilian Society of Urology. The survey included an assessment of socio-demographic, clinical practice, educational, health-related and behavior parameters. We also evaluated which subareas of urology were predominantly affected. A similar survey was adapted and sent to the directors of all urology residency programs.

**Results:**

COVID-19 pandemic has severely impacted the clinical, surgical, and educational activities of urology residents in Brazil. Urology residents reported >50% decrease in multiple surgical modalities. We highlight kidney transplantation surgeries (66.2%), minor surgeries (62.3%), endoscopic surgeries (42.6%) and reconstructive surgeries (38.8%). This could represent a critical skills gap that residents may face beyond the COVID-19 pandemic. Furthermore, PG students faced stressful situations that caused worsening of mental and physical health, such as getting redirected to assistance of COVID-19 patients (66.9%), and high rate of infection by SARS-CoV-2 (58.2%).

**Conclusions:**

The COVID-19 pandemic has severely impacted the clinical, surgical, and educational activities of urology residents in Brazil. This could represent a critical skills gap that residents may face beyond the COVID-19 pandemic. PG students faced stressful situations that caused worsening of mental and physical health such as redirection to assistance of COVID-19 patients, concern about their own contamination and of family members.

## INTRODUCTION

The emergence of the COVID-19 pandemics transformed the medical assistance all around the World. It led to a great reduction of medical consultations, diagnostic evaluations and surgeries of any kind ( 1 , 2 ). While the pandemics has subsided, it has left enduring sequelae in the health system and profound impacts on medical training ( 3 , 4 ).

Urology residents have dealt with major challenges not only in terms of medical training, but also regarding their personal lives, health and well-being ( 5 ). There was a great decrease in hands-on urological activities such as elective consultations and surgeries, as well as educational activities such as general meetings, classes and journal club discussion ( 6 , 7 ). In addition, social distancing has caused changes in the residents’ lifestyle. Many have been experiencing anxiety disorders and feeling of exhaustion ( 8 – 10 ). Compensation for educational damage has been inconsistent and there is a need to understand the real magnitude of this damage and other impacts on medical residence. Most studies on this subject evaluated the short-time impact of COVID-19 and the long-term impact remains unknown.

In Brazil, COVID-19 started in March/2020, which coincided with the start of the new residency year. In this study, we evaluated the impact of COVID-19 on a complete year of urology residency training. Our main hypothesis is that the COVID-19 pandemic had a profound impact on the training of urology residents due to the decrease in surgical procedures and theoretical educational activities. Furthermore, we believe that this sub-group of healthcare professionals had higher rates of SARS-CoV-2 infection than their peers and significant harm to mental health. The primary goal was to evaluate clinical practice and urological training during the whole year of residency they had just completed under the COVID-19 pandemics (March/2020 to February/2021). Secondary goals involved assessing residents' infection rate and health parameters. We also evaluated the opinion of the directors of urology programs on these topics.

## MATERIAL AND METHODS

An electronic survey was e-mailed in June/08/2021 to all postgraduate students (PGY 3 to 5) from official urology residency programs (URP) registered by the Brazilian Society of Urology, during the academic year starting in March/2020. Data collection was closed on July/04/2021. The invitation e-mail contained a link to a 46-question, web-based survey ( Supplementary material 1, see more ). All questions were closed-ended, multiple choice. The survey included an assessment of socio-demographic, clinical practice, educational, health-related and behavior parameters. A similar survey was adapted and sent to the directors of all URPs. The invitation e-mail contained a link to a 27-question web-based inquiry ( Supplementary material 2, see more ). The questionnaire addressed many of the same points evaluated by the residents, from the perspective of the program director.

**Volume of medical activities and impact on different urological subareas:** We assessed the volume of consultations, exams and surgical procedures, but also the resident's perception of prejudice on their training in each urological sub-area. We also assessed urology resident's deployment to the front-line treatment of COVID-19 patients and the availability of personal protective equipment.

**Impact on educational activities:** We evaluated the impact of the pandemics on in-person educational activities, such as classes, clinical meetings and grand round discussions and evaluated the new formats of online urological training implemented by the residency programs. Residents were asked about their preferences regarding online urological education and additional training offered by their residency program to compensate for the disturbed education.

**Impact of COVID-19 pandemics on urology residents’ health:** We investigated the rate of SARS-CoV-2 infection among urology residents and the severity of the disease. We also evaluated health parameters and lifestyle changes during the studied period, including weight gain, physical activities, alcoholic beverages consumption, sexual activity, satisfaction with general health, depressive symptoms and feeling of exhaustion.

### Data collection and Statistical analyses

Data were initially elaborated using Survey Monkey® software online. Quantitative variables were expressed as medians and interquartile ranges, while qualitative variables were expressed as absolute values, percentages, or proportions.

Student's t or ANOVA was used to compare continuous variables. Categorical variables were compared using the Chi-squared or Fisher's exact test. Associations were described as Odds Ratios with respective confidence intervals. All tests were 2-sided and a p value < 0.05 was considered statistically significant. GraphPad Prism, version 8.0.4, San Diego-CA, USA, was used for data analysis.

## RESULTS

A total of 157 urology residents completed the survey, representing 33.5% of all the residents in the country. Most respondents (89.1%) were men, and the median age was 31 (± 3) years. Participants were 37 (23.5%) PGY-3 residents, 55 (35.0%) PGY-4 and 65 (41.4%) PGY-5 residents. The distribution of participants was proportional to the actual distribution of Brazilian urology residents across the country's five geographic regions. São Paulo State accounted for 37.1% of participants.

Most participants (82.8%) attend a URP in a public hospital and most respondents (82.0%) stated that their hospital was transformed in a referral center for COVID-19 patients, with a very high volume of admissions throughout most of the 12-month period of the study.

### Impact on medical activities of different urological subareas

Table-1 shows the impact of one year of COVID-19 on the volume of various urological clinical and surgical activities in comparison to the pre-pandemic year. All activities were significantly reduced. A reduction of >50% was reported by most participants in kidney transplant surgery (66.2%); minor surgeries (62.3%) (i.e., vasectomy, circumcision, hydrocelectomy) and urodynamic testing (53.4%). Areas that were least affected included emergency consultations (20.3%), major oncologic surgeries (25.4%), and endoscopic surgeries for lithiasis (28.5%).

**Table 1:** Impact of one year of COVID-19 on urology residents’ practice.

Practice activity		%
**Elective patient visits**		
	Remained stable	10.8
	Decreased up to 25%	21.6
	Decreased 25 to 50%	31.2
	Decreased 50 to 75%	25.4
	Decreased > 75%	10.8
**Emergency patient visits**		
	Remained stable	33.1
	Decreased up to 25%	26.7
	Decreased 25 to 50%	19.7
	Decreased 50 to 75%	15.9
	Decreased > 75%	4.4
**Minor surgeries (i.e. vasectomy, circumcision, hydrocelectomy)**		
	Remained stable	5.7
	Decreased up to 25%	12.7
	Decreased 25 to 50%	19.1
	Decreased 50 to 75%	22.9
	Decreased > 75%	39.4
**Endoscopic surgeries (i.e. TURP** * **, TURB** ** **)**		
	Remained stable	7.6
	Decreased up to 25%	20.3
	Decreased 25 to 50%	29.3
	Decreased 50 to 75%	25.4
	Decreased > 75%	17.2
**Endoscopic lithiasis surgery (i.e. ureterolithotripsy)**		
	Remained stable	26.7
	Decreased up to 25%	23.5
	Decreased 25 to 50%	21.0
	Decreased 50 to 75%	15.2
	Decreased > 75%	13.3
**Major oncologic surgeries**		
	Remained stable	24.8
	Decreased up to 25%	22.9
	Decreased 25 to 50%	26.7
	Decreased 50 to 75%	14.6
	Decreased > 75%	10.8
**Reconstructive surgeries (38.8%)**		
	Remained stable	24.8
	Decreased up to 25%	11.4
	Decreased 25 to 50%	24.8
	Decreased 50 to 75%	19.1
	Decreased > 75%	19.7
**Kidney Transplants**		
	Remained stable	7.0
	Decreased up to 25%	8.2
	Decreased 25 to 50%	18.4
	Decreased 50 to 75%	24.8
	Decreased > 75%	41.4
**Diagnostic procedures (Cystoscopies)**		
	Remained stable	18.5
	Decreased up to 25%	18.5
	Decreased 25 to 50%	32.6
	Decreased 50 to 75%	18.5
	Decreased > 75%	11.5
**Urodynamic Testing**		
	Remained stable	10.8
	Decreased up to 25%	18.4
	Decreased 25 to 50%	17.2
	Decreased 50 to 75%	19.7
	Decreased > 75%	33.7

* TURP: Transurethral resection of prostate;

** TURB: Transurethral resection of bladder tumor

**Figure 1:**
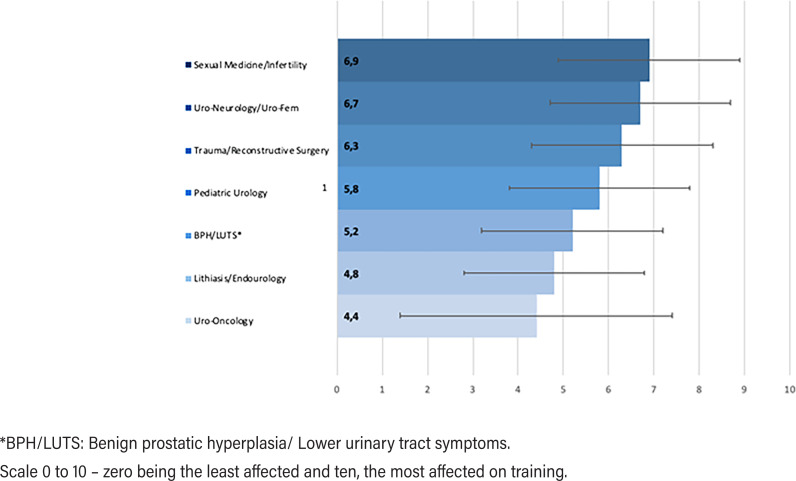
Resident's perception of adverse effects in the training of different urological subspecialties (higher scores indicate worse prejudice).

Figure-1 depicts the resident's perception of prejudice on their training in each urological subspecialty assessed with a visual analog scale ranging from 0 to 10 (0 being the least prejudice). Uro-oncology was the least affected subspecialty (4.4 ± 2.9), followed by lithiasis (4.8 ± 2.8). The areas considered with worst prejudice on training were sexual medicine/andrology (6.9 ± 2.9) and female urology/neuro-urology (6.7 ± 2.7).

PGY-5 were considered, by all groups combined, as those with the greatest educational damage (47.7%). Interestingly, however, PGY-5 signaled that the most harmed group was PGY-4.

Most residents (66.9%) were relocated to work in the front-line treatment of COVID-19 patients, at some point, during the evaluated period. Regarding the availability of personal protective equipment (PPE): 53 (47.7%) reported shortage of N-95 masks and 51 (45.9%) of waterproof aprons.

### Impact on educational activities

Many scientific and educational activities were cancelled during this period. Bedside clinical rounds (49.0%) and urology department meetings (46.5%) were the two activities that were more frequently cancelled in the period. Only 28 (17.8%) residents claimed that their URP did not cancel any activity. Several smart learning modalities and online meetings and contents were developed.

In our cohort, 116 (73.9%) and 95 (60.5%) urology residents mentioned the general urology department meeting and clinical cases discussion as the most implemented online tools by their URP. Furthermore, 119 (76.3%) urology residents attended regular webinars focused on clinical cases and journal clubs; and 94 (60.2%) watched on-line lectures.

Regarding the intention to supplement urological training after the end of the residency program, 40 (61.5%) PGY-5 declared they would like to pursue fellowships in some subspecialty area and 28 (43.0%) would like to take short training periods in focused sub-areas. Only 6 (9.2%) stated they had no interest in further training.

Most residency programs (89.0%) did not offer alternatives to supplement urology training after the end of the year. As a consequence, most residents (74.2%) were mostly dissatisfied with the lack of actions proposed by the program directors.

### Impact of COVID-19 pandemics on urology residents’ health

Ninety-two (58.6%) respondents claimed to have had COVID-19 infection in the studied period, including 45.9% with unequivocal laboratory confirmation and 12.7% with a clinical diagnosis. Clinical presentation was mild or moderate in all cases with only one respondent reporting the need for hospitalization for a few days. The impact of COVID-19 on urology residents’ health parameters is shown in Table-2 . Most residents considered themselves satisfied (41.9%) or very satisfied (11.6%) with their general health, while 25.7% were unsatisfied. Sadness or depressive feelings were reported as usual by 22.3% of the participants while exhaustion was reported by 48.7%. The comparison between residents of different years did not result in differences regarding health-related parameters.

**Table 2:** Changes in urology residents’ health parameters during the first year of COVID-19.

Health parameters		%
**Weight**		
	Reduced	22.3
	Stable	31.8
	Increased	45.9
**Physical activity**		
	Reduced	58.6
	Stable	19.1
	Increased	22.3
**Alcoholic beverages intake** *		
	Reduced	9.2
	Stable	53.2
	Increased	37.6
**Sexual activity**		
	Is worse	18.6
	Is stable	64.7
	In better	16.7
**Satisfaction with own general health**		
	Very unsatisfied	11.6
	Unsatisfied	14.1
	Not satisfied nor unsatisfied	20.6
	Satisfied	41.9
	Very satisfied	11.6
**Frequency of sadness or depressive feelings**		
	Never	8.9
	Rarely	36.9
	Sometimes	31.9
	Usually	16.6
	Very usually	5.7
**Feeling of exhaustion**		
	Never	2.5
	Rarely	15.5
	Sometimes	33.3
	Usually	32.0
	Very usually	16.7

* Participants who reported not drinking alcoholic beverages were removed from calculations

### Impact of COVID-19 according to the urology residency directors

A total of 58 URP directors completed the survey, representing 74.3% of all programs in the country. They confirmed major reductions of various urological clinical and surgical activities in comparison to the pre-pandemic year.

The magnitude of reduction estimated by directors was similar to the residents’ perceptions for elective and emergency consultations, urodynamics, cystoscopies, minor surgeries and endoscopic prostate surgeries. However, the directors diverged from the residents and estimated a lesser degree of reduction of ureteroscopies, oncologic surgeries, reconstructive surgeries and kidney transplantation ( Table-3 ).

**Table 3:** Comparison between residents and directors regarding the decrease in the volume of surgeries and consultations in URPs.

	RESIDENTS		DIRECTORS		
	< 50% reduction	> 50% reduction	< 50% reduction	> 50% reduction	P VALUE
Elective Consultations	100	57	45	15	0.146
Emergency Consultations	125	32	53	08	0.417
Urodynamics	73	84	27	34	0.879
Cystoscopies	109	48	38	22	0.419
Minor Surgeries	59	98	24	36	0.757
TURP *	90	67	41	19	0.163
Ureteroscopies	112	45	52	09	0.036
Oncologic Surgeries	117	40	53	05	<0.001
Reconstructive Surgeries	53	104	29	27	<0.001
Kidney Transplants	96	61	28	23	<0.001

* TURP: transurethral resection of the prostate.

Directors' perception of residents training damage in pediatric urology, BPH/LUTS, sexual medicine/infertility and neurourology was similar to the residents' perception. Directors estimated a lesser degree of training harm in uro-oncology and lithiasis/endourology ( Table-4 ). Among URP directors, 45.0% considered the PGY-4 as the most impacted trainees, 31.7% the PGY-5, and 23.3% the PGY-3.

**Table 4:** Comparison between residents and directors regarding their evaluation of harm in urologic training in different sub-areas.

		RESIDENTS	DIRECTORS	
		Mean	Mean	P VALUE
**Lithiasis/Endourology**		**4.8 (± 2.8)**	**3.5 (± 2.3)**	**0.003**
	BPH/LUTS *	5.2 (± 2.9)	5.0 (± 2.3)	0.654
	Sexual Medicine/Infertility	6.9 (± 2.9)	6.8 (± 2.3)	0.517
	Trauma/Reconstructive Surgery	6.3 (± 2.9)	5.8 (± 2.4)	0.255
**Uro-Oncology**		**4.4 (± 3.0)**	**3.2 (± 2.0)**	**0.013**
	Uro-Neurology/Uro-Fem	6.7 (± 2.7)	6.5 (± 2.2)	0.280
	Pediatric Urology	5.8 (± 3.3)	6.1 (± 2.8)	0.636

* BPH/LUTS: Benign prostatic hyperplasia/ Lower urinary tract symptoms.

Scale 0 to 10 – zero being the least prejudice and ten, the most prejudice.

## DISCUSSION

This study showed the heavy impact of the pandemics' restrictions on urology residents’ education and clinical practice. We observed a great decrease in the volume of outpatient visits and elective surgeries. In addition, residents reported negative consequences for their health and well-being, with a great proportion reporting weight gain (45.9%), reduction of physical activities (58.6%), and increasing alcohol intake (37.6%). Mental health was an important issue as well, feeling of sadness or depression (22.3%) and feeling of exhaustion (48.7%) were present in a considerable proportion of respondents. Remarkably, 58.6% of the residents contracted COVID-19.

The study took place in June 2021, 15 months after the onset of the pandemic, with participants commenting on the period from March 2020 to March 2021. This time frame marked the first year of the pandemic, characterized by strict social and economic restrictions. Brazil was particularly hard-hit, ranking second in deaths and third in infections due to COVID-19 throughout this period ( 11 ).

In this study, we had 157 participants, representing 33.5% of the 468 eligible urology residents. We were hoping to have a higher participation rate. A previous study assessing the short-term impact of the pandemic on Brazilian urology residents achieved a 58.7% response rate ( 10 ). We believe that the physical and emotional fatigue associated with the pandemics restrictions and the fact that the questionnaire was long may have contributed to the lower participation rate in this survey. However, our participation rate aligns other similar surveys, ranging from 15% to 60.8% ( 5 , 9 , 12 ). Most participants were PGY-5 residents (41.40%), followed by PGY-4 (35.03%) and PGY-3 (23.57%) residents. We hypothesize that senior residents were more inclined to participate due to heightened concerns about the pandemic's impact on their training.

Residents reported a substantial reduction in all clinical and surgical activities. Similar results have been observed globally. In Turkey, an online survey assessed the impact of the pandemics on functional urology practice, and found a decrease in outpatient clinics, urodynamic testing and elective surgeries ( 13 ). Comparable findings were reported in Italy ( 14 ), aligning with urological society guidelines that recommended prioritizing more urgent diseases, the postponement of which could affect cure chances ( 15 , 16 ).

The pandemic led to the cancellation of most educational and scientific activities. Most of the residents made use online smart learning tools, such as discussion-focused webinars (76.3%) and pre-recorded on-line lectures (60.2%). These findings are in line with other countries. In Italy, 38.8% of urology residents utilized webinars for smart learning ( 9 ). In Indonesia, web-based video conference was the most used method educational activity during the pandemic ( 16 ). A survey by the American Confederation of Urology (CAU) showed that 93% of residents attended webinars during the pandemic ( 17 ).

COVID-19 restrictions have impacted people's lifestyles around the World. There were reports of decreased physical activity, weight gain, and increased alcohol and tobacco consumption ( 18 , 19 ). Brazilian physicians reported similar effects ( 4 , 10 ). In the present study, 58.6% of the residents reported reduced physical activity, and 45.8% reported weight gain throughout the one-year study period. Over 25% of the participants were dissatisfied with their general health. Additionally, 48.7% had a feeling of exhaustion and 21.4% reported recurrent sadness and depressive feelings. These findings indicate a significant impact on overall well-being and mental health which is very concerning. Residents from different PGYs were similarly affected. Our findings align with other studies reporting mental health problem rates ranging from 33 to 57.6% ( 20 , 21 ). A systematic review of 33,062 frontline health care workers, found a pooled prevalence rate of anxiety of 23.2%, and depression prevalence of 23.8% ( 22 ). Burnout among physicians is strongly associated with the career disengagement, suboptimal patient care and patient safety incidents ( 23 ).

Our findings indicate that 58.6% urology residents contracted COVID-19, which is an exceptionally high infection rate among Brazilian urology residents during the pandemic's first year. Contrastingly, only 4.71% of the Brazilian general population were infected with SARS-COV-2 at the same period ( 11 ). Globally, COVID-19 rates in medical residents varied from 5.0% to 26.3% at different times.

In our study, 105 (66.8%) residents were redeployed to work with patients infected with COVID-19. In Spain, 50% of urology residents were recruited to COVID-19 specific units ( 24 ). In the United States, urology program directors reported that 26% of residents were shifted to treat COVID-19 patients ( 5 ). A key concern regarding our residents was workplace exposure and appropriate PPE availability. Nearly half of the participants reported a lack of N-95 masks (47.7%), waterproof aprons (45.9%), protective goggles (42%) at their hospitals. Comparable outcomes were noted in New York, where the practice of mask reuse was documented ( 25 ), and in France, where 43% of residents reported inadequate access to PPE ( 19 ).

Most (93%) PGY-5 residents expressed an intention to complement their training after finishing urology residency. US studies showed significant concern among residents and URP directors regarding the impact of COVID-19 in medical training, including failure to meet clinical visit and surgery goals and fearing a lack of skill for a fellowship or future job ( 26 , 27 ). Most URPs did not present a plan to mitigate the training damage. Despite the uniformity of prejudice in urology training across multiple countries, there were no effective compensatory strategies ( 9 , 28 ).

This study's primary strength lies in its evaluation of urology residents after one year of the pandemic, coinciding with a full year of residency. Participants were evenly distributed across various postgraduate levels and represented all five geographic regions of the country. We conducted a comprehensive assessment of the pandemic's impact on a full year of urology residency training, covering medical practice, educational activities, and health and lifestyle parameters. Additionally, we identified which subareas of urology were predominantly affected based on surgery volume and residents’ perceptions of prejudice. The study provides insight into URP directors’ views on resident training. A notable limitation of the study is the length of the questionnaire, which may cause participants to become bored while completing it. Additionally, many of the instruments used to evaluate various parameters are not validated. For example, mental health was assessed with a single question instead of using a validated questionnaire. Another limitation is the requirement for participants to compare their current state with the previous year, which could introduce recall bias. Further research should confirm these findings across different medical specialties to develop strategies for mitigating training losses. This study sheds light on the challenges faced by urology postgraduate students during the pandemic.

## CONCLUSIONS

The COVID-19 pandemic has severely impacted the clinical, surgical, and educational activities of urology residents in Brazil, regardless of the residency year. PG students faced stressful situations that caused worsening of mental and physical health such as redirection to assistance of COVID-19 patients, concern about their own contamination and of family members and shortages in PPE, in addition of the aforementioned educational loss. This could represent a critical skills gap that residents’ may face beyond the COVID-19 pandemic. The program directors and entities responsible for the quality of medical training must assess the difficulties imposed by the pandemic and formulate a compensation plan to try to soften the impact on training residents. Future research, with a longer follow-up time will be needed to accurately measure the impact of this pandemic on urology training.
